# Exploring an Innovative Approach: Integrating Negative-Pressure Wound Therapy with Silver Nanoparticle Dressings in Skin Graft Procedures

**DOI:** 10.3390/jpm14020206

**Published:** 2024-02-14

**Authors:** Abdalah Abu-Baker, Andrada-Elena Țigăran, Teodora Peligrad, Daniela-Elena Ion, Daniela-Elena Gheoca-Mutu, Adelaida Avino, Cristian-Sorin Hariga, Oriana Elena Moraru, Laura Răducu, Radu-Cristian Jecan

**Affiliations:** 1 Doctoral School, “Carol Davila” University of Medicine and Pharmacy, 010221 Bucharest, Romania; abdalah.abu-baker@drd.umfcd.ro (A.A.-B.); adelaida.avino@drd.umfcd.ro (A.A.); 2 Department of Plastic Surgery, “Prof. Dr. Agrippa Ionescu” Emergency Clinical Hospital, 011356 Bucharest, Romania; andrada-elena.tigaran@rez.umfcd.ro (A.-E.Ț.); teodora.peligrad@rez.umfcd.ro (T.P.); daniela-elena.ion@rez.umfcd.ro (D.-E.I.); cristian.jecan@umfcd.ro (R.-C.J.); 3 Discipline of Anatomy, “Carol Davila” University of Medicine and Pharmacy, 010221 Bucharest, Romania; 4 Department of Plastic Surgery, Emergency Clinical Hospital, 014461 Bucharest, Romania; 5 Discipline of Plastic Surgery, “Carol Davila” University of Medicine and Pharmacy, 010221 Bucharest, Romania; 6 Discipline of Cardiovascular Surgery, “Carol Davila” University of Medicine and Pharmacy, 010221 Bucharest, Romania; orianaelenamoraru@umfcd.ro; 7 Department of Vascular Surgery, “Prof. Dr. Agrippa Ionescu” Emergency Clinical Hospital, 011356 Bucharest, Romania

**Keywords:** chronic wounds, silver nanoparticles, negative wound pressure therapy, wound healing

## Abstract

Background: Skin grafting is a helpful instrument in a plastic surgeon’s arsenal. Several types of dressings were designed to facilitate the process of graft integration. Negative-pressure wound therapy is a proven dressing method, enhancing graft survival through several mechanisms: aspiration of secretions, stimulation of neoangiogenesis, and promotion of an anti-inflammatory environment. Silver nanoparticle dressings also bring multiple benefits by bearing an antimicrobial effect and providing a humid medium, which are favorable for epithelialization. The combination of NPWT (negative-pressure wound therapy) with AgNPs (silver nanoparticles) has not been widely studied. Materials and methods: This study aimed to compare the outcomes of silver nanoparticle sheets with the combination of negative-pressure wound therapy and silver nanoparticle dressings. We conducted a comparative prospective study on 80 patients admitted to the Plastic Surgery Department of “Prof. Dr. Agrippa Ionescu” Emergency Clinical Hospital between 1st of January 2020 and 31st of December 2022. The study population was randomized to receive either silver nanoparticle dressings or negative-pressure wound therapy (NPWT) combined with silver nanoparticle dressings. Various parameters were monitored, including patient comorbidities and graft-related data such as defect etiology, graft integration, and graft size. Dressings were changed, and graft status was evaluated at 7, 10, and 14 days postoperatively. Additionally, baseline C-reactive protein (CRP) levels were measured before surgery and 7, 10, and 14 days postoperatively. Results: The study demonstrated an enhanced integration of skin grafts at all evaluation stages when employing NPWT combined with AgNPs, particularly evident 10 days post operation. Significant variations in graft integration were also observed based on factors such as diabetes, cardiovascular disease, graft size, or the origin of the grafted defect. Moreover, dynamic C-reactive protein monitoring showed a statistically significant decrease in CRP levels 10 days post operation among patients treated with NPWT in conjunction with silver dressing, consistent with the nearly complete integration of skin grafts at this evaluation threshold. Conclusion: Several factors influence the postoperative evolution of split-skin grafts. Postoperative dressings target local factors to enhance graft integration further. Our research demonstrated that the innovative combination of NPWT-assisted dressings, complemented by a silver nanoparticle sheet, resulted in improved benefits for graft integration and the alleviation of systemic inflammation.

## 1. Introduction

An essential and valuable tool in a plastic surgeon’s reconstructive toolkit is skin grafting. It provides a quick and straightforward method to cover a variety of defects, including burns, chronic skin ulcers, and post-excisional soft-tissue defects, which would typically require a longer period to heal through secondary epithelialization [1]. Skin grafting involves the excision of an area of epidermis with a variable amount of the underlying dermis, applied to an open wound [2].

Grafts generally integrate well into healthy wound beds. However, several postoperative circumstances have a significant impact on integration. For instance, the presence of fluid between the wound bed and the graft can lead to graft separation and hinder the establishment of new blood supply [3]. Hemostasis must, therefore, be carefully performed during surgery to avoid hematomas. Seromas underneath the graft are better tolerated than hematomas but can lead to graft necrosis nonetheless [4]. A practical solution to fluid build-up involves creating tiny pores in the graft surface to facilitate drainage [5]. Infection is another factor that impedes graft integration, evident through local and systemic inflammatory responses. Graft movement in relation to the bed may also result in a lack of graft integration because the freshly generated capillaries are unable to inosculate the graft [6].

To counteract these undesirable events, various types of dressings were designed [7]. These dressings aimed to mitigate fluid build-up and prevent graft movement by compressing the graft onto the wound bed while also inhibiting bacterial proliferation [8]. Additionally, some of these dressings can modulate the local wound environment at the molecular level, favoring healing-prone conditions, which, in turn, lead to an improved graft take.

Negative-pressure wound therapy (NPWT) stands as a revolutionary tool in plastic surgery, offering extensive benefits for wound healing. NPWT is utilized to treat various disorders, including acute wounds, posttraumatic deformities, chronic ulcers, and skin grafts [9]. The mechanism typically involves placing a sterile sponge directly on top of the wound, covered with an impermeable sheet, to create a hermetically sealed area. Subsequently, a negative pressure pump is connected to the newly created compartment, subjecting the dressed area to a vacuum [10].

Multiple publications support the role of NPWT in wound healing. Usually, we can find an overexpression of TNFα (tumor necrosis factor α), interleukin-1, and interleukin-6 in chronic wounds, originating from hyperglycemia and persistent wound inflammation. High concentrations of such cytokines promote a proinflammatory milieu that inhibits angiogenesis and proper wound healing [11]. NPWT application produces a substantial decrease in early local tumor necrosis factor (TNF) and interleukin (IL) 1 expression for both chronic and acute wounds [12,13]. As well as for diabetic wounds, NPWT was found to be effective in suppressing the IL-6 expression, therefore enhancing the healing process [14,15].

Potent proangiogenic and chemokine IL-8 is also involved in controlling neutrophil and macrophage migration, thus hastening wound healing [16,17]. The application of NPWT resulted in a significant increase in local IL-8 expression in a study involving human wounds [15]. Within four hours of applying NPWT, there was a notable increase in systemic levels of the anti-inflammatory cytokine IL-10 [14].

Another revolutionary tool is silver nanoparticle dressings. Silver nanoparticles exert a significant antimicrobial effect upon contact with tissues. In its metallic state, silver is inert and lacks biocidal properties. However, in the presence of water or tissue fluids, it ionizes, releasing Ag+ or other physiologically active ions. This “activated” ion has a high affinity for sulphydryl groups and protein residues on cell membranes [18]. Importantly, silver exhibits antibacterial activity even at concentrations that are as low as 1 ppm. Silver particles accumulate in sensitive microorganisms against a concentration gradient, ultimately leading to cellular death [19].

AgNPs (silver nanoparticles) also influence factors related to both growth and inflammation. AgNP dressings were shown to produce a significantly higher concentration of VEGF (vascular endothelial growth factor). These dressings can also increase the level of other growth factors, such as TGF-β1(transforming growth factor β1) and bFGF (fibroblast growth factor), as well as reduce the levels of pro-inflammatory markers such as TNF-α and IL-1β. Due to their effect on the inflammatory response, AgNPs are considered highly beneficial in the wound healing process [20].

While the literature has extensively discussed the advantages of using NPWT and silver dressings separately, there is limited information available on their combined usage in the management of skin grafts. This study aimed to demonstrate that the combination of silver dressings with NPWT offers a beneficial approach to managing lower-extremity grafts.

## 2. Materials and Methods

This study aimed to compare the effects of tie-over silver nanoparticle dressings and NPWT used in combination with the AgNP dressings on graft integration. We conducted a clinical observational prospective study involving 80 patients hospitalized in the Plastic Surgery Department of “Prof. Dr. Agrippa Ionescu” Emergency Clinical Hospital between 1st of January 2020 and 31st of December 2022 with lower-limb-skin and soft-tissue defects. Informed consent was obtained from all subjects involved in the study.

### 2.1. Inclusion Criteria

- Patients with lower-limb soft-tissue defects that required grafting;
- Adults.

### 2.2. Exclusion Criteria

- Children;
- Pregnant women;
- Patients who refused to participate;
- Patients discharged earlier than 14 days;
- Active infections.

### 2.3. Variables and Definitions

The data collected included patient-dependent variables, such as sex, age, cardiovascular and diabetic comorbidities, as well as graft-related factors: percentage of graft take 7, 10, and 14 days post operation, graft size, and defect origin. Grafted defects were acquired either through ulceration debridement or excision of a soft tissue mass.

Graft status was assessed through a clinical evaluation of integration signs, including color, graft aspect, level and quality of exudate, and visualization of epithelium between the graft net branches. We calculated the percentage of graft integration by dividing the viable graft area by the total surface of the graft.

The graft area was measured in cm^2^. The cohort was then divided based on graft area in three categories:

- Graft area smaller than 150 cm^2^–25 patients;
- Graft area between 150 cm^2^ and 500 cm^2^–42 patients;
- Graft area greater than 500 cm^2^–13 patients.

We also measured C-reactive protein (CRP) levels before surgery and 7, 10, and 14 days post operation. Thus, we were able to evaluate the CRP levels dynamically and compare their evolution depending on the type of dressing used.

The study population was randomized to receive either of the proposed dressing methods.

Out of 80 patients, 41 were treated with silver nanoparticle compressive dressings. This dressing consisted of an AgNP sheet placed over the graft and secured with a tie-over bolster, providing pressure and preventing graft movement. The dressing was changed 7, 10, and 14 days post operation for local lavage and to assess graft status.

**Figure 1:**
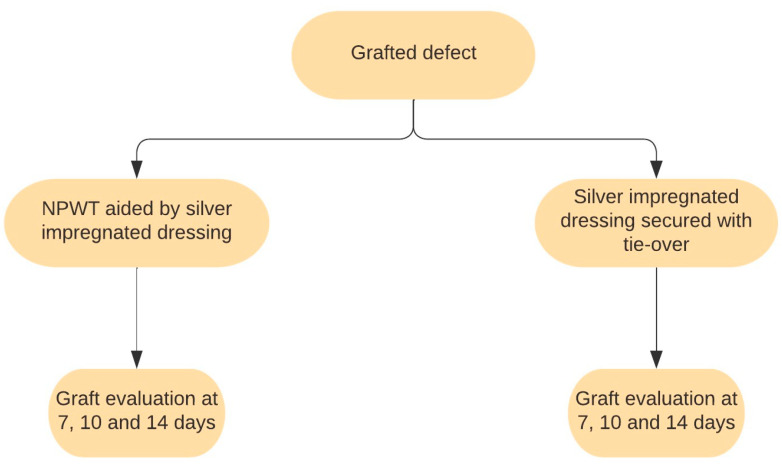
Scheme of dressing methods and schedules.

The other 39 patients benefited from NPWT aided by silver nanoparticle dressing. The first layer consisted of an AgNP dressing directly applied to the skin graft. The second layer comprised the NPWT dressing connected to the vacuum machine. The negative-pressure pump operated on an alternating regime of 125 mmHg for 3 min, followed by 75 mmHg for another 3 min. The NPWT dressing was changed 7, 10, and 14 days post operation, and the graft status was assessed. Figure 1 illustrates the dressing and assessment schedule.

### 2.4. Statistical analysis

Data collection was performed using Microsoft Office Excel 2010. We performed statistical analysis using SPSS version 26.0 software (IBM Corporation, Armonk, NY, USA), all taking into consideration a 95% confidence interval. Text editing was performed using Microsoft Office Word 2010.

## 3. Results

The study population comprised 44 males and 36 females, with a mean age of 64 years. Among the participants, 58 had cardiovascular comorbidities, and 61 had type 2 diabetes mellitus. Regarding the origin of the defects, 28 resulted from the excision of soft-tissue tumors, while the remaining 52 followed chronic ulcer debridement. The cohort included 25 patients with small grafts (<150 cm^2^), 42 patients with medium-sized grafts (150–500 cm^2^), and 13 patients with large grafts (>500 cm^2^). The distribution of the subjects into the two study lots is illustrated in Table 1.

Comparing the two methods of graft dressing on the 7, 10, and 14 days post operation, we observed a higher graft integration in patients who received the combination of NPWT and AgNP-impregnated sheets. The average graft take at the 7th day for grafts dressed with only a silver layer was 74.88%, compared to 85% for those treated with NPWT and AgNP dressing. On the other hand, at the 10-day threshold, a significant difference was observed between the two means, with a graft take of 87.20% for silver nanoparticle sheets and 97.56% for vacuum-assisted dressings combined with AgNPs. This difference persisted until 14 days post operation, with a mean integration of 93.41% for silver-dressed grafts and 99.62% for grafts treated with NPWT and AgNPs. We can observe a better result in graft integration for the NPWT+AgNP lot, especially at the 7- and 10-day thresholds. All these differences were statistically evaluated using the independent T-test, which showed a high degree of statistical significance (*p* < 0.001). The statistical data are also displayed in Table 2.

We assessed the graft evolution separately depending on the presence of cardiovascular disease. We evaluated the effect size of applying NPWT+AgNPs using the Cohen D formula. The data obtained are available in Table 3. Our observations reveal that the combination of NPWT and silver nanoparticle dressings resulted in improved graft integration at every evaluation threshold, showing a statistically significant difference for patients with cardiovascular disease (*p* < 0.001). The data also indicate a strong effect size at every threshold for patients with cardiovascular comorbidities (D < −0.8), with the most substantial difference observed one week after grafting.

Regarding the presence of diabetes mellitus, we observed an improvement in graft integration with the use of NPWT+AgNPs. The independent *T*-test showed a statistically significant difference in graft take when adding NPWT to the dressing (*p* < 0.001) for diabetic patients. We also observed a strong effect size (D < −1) for diabetic patients at all stages. Data is presented in Table 4.

Depending on graft area, we separated the patients into three categories. We observed that the NPWT+AgNP dressings resulted in higher graft integration rates for every category compared to simple silver nanoparticle dressings. Moreover, this improvement was statistically significant for medium and large grafts (*p* < 0.01), as illustrated in Table 5.

Taking into consideration the effect size of NPWT+AgNPs, we observed a varied D coefficient for small grafts (ranging from −0.46041 to −0.77274), a strong D coefficient (D < −0.8) for medium graft, and an even stronger D coefficient for large grafts. These data show that the benefit of choosing NPWT+AgNPs is far greater for grafts of a larger area compared to smaller grafts.

The defect etiology, whether resulting from the excision of a soft-tissue tumor or the debridement of an ulceration, may influence the integration process of the graft. Post-excisional defects typically represent clean surgical wounds, whereas debrided ulcerations may still carry inflammatory and infectious repercussions specific to chronic wounds.

The data analysis showed, once again, an improvement in graft integration for patients treated with NPWT+AgNPs. According to our study, this progress was statistically significant (*p* < 0.001) at the 7- and 10-day thresholds. The effect size generally showed a strong benefit resulting from using silver dressings aided by NPWT, even if there was no clear distinction between the two categories. Statistical data analysis is shown in Table 6.

**Figure 2:**
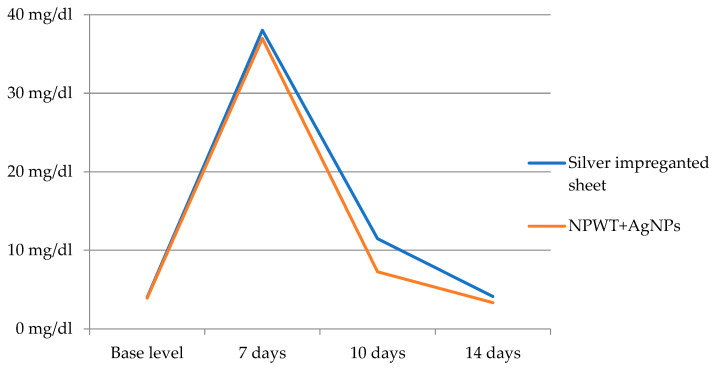
Dynamic variation of CRP levels.

The measurements of the C-reactive protein showed similar starting points before grafting. At the 7-day evaluation, a small difference was observed between the two lots, with an average of 36.97 mg/dL for the NPWT+AgNPs group compared to 38.02 mg/dL for the patients with silver dressings. However, the difference was not statistically significant. A considerable decrease in the CRP level for patients who received NPWT+AgNP dressings was observed on day 10 after grafting, with a level of 7267 mg/dL, compared to 11,473 mg/dL for the other lot, as demonstrated in Table 7. Dynamic monitoring illustrated a tendency towards the normalization of CRP levels 14 days post operation, with the difference between the two groups decreasing (3338 mg/dL for patients dressed with NPWT+AgNPs compared to 4129 mg/dL for the group dressed with a silver sheet). It should be noted that the CRP values 14 days after the grafting were close to the baseline values measured before the surgical intervention. From this point of view, only the patients belonging to the group dressed with NPWT aided by silver nanoparticle sheets had lower CRP serum levels than the post-operation levels. We performed the statistical analysis of the data related to the C-reactive protein level and noted a statistically significant difference at the 10-day evaluation, *p* = 0.001. The dynamic variation of CRP levels is also displayed in Figure 2.

## 4. Discussion

As expected, NPWT systems confer a considerable benefit for skin graft integration, directly addressing most of the possible causes of failure, while enhancing graft revascularization. Compared to conventional skin graft dressings, NPWT was superior in most aspects, with higher graft integration rates, especially at the earlier evaluation checkpoints.

Negative pressure creates multiple benefits for skin graft integration, such as removing excess fluid build-up from the wound bed and contracting the wound edges by a mechanical force [21]. Additionally, the negative pressure generated by the vacuum exerts effects at the molecular level, inhibiting proinflammatory chemokines and promoting the secretion of anti-inflammatory and angiogenic growth factors, such as interleukin-10 and vascular endothelial growth factor [22]. Thus, NPWT is responsible for creating a healing-prone medium supporting neo-angiogenesis and epithelialization [23]. Another advantage of vacuum devices is minimizing the bacterial population of an infected wound [24]. Moreover, vacuum-assisted dressings ensure a lack of movement between the graft and the wound bed by applying pressure evenly on all sides of the wound [25]. These effects can be achieved while the patients maintain a certain degree of mobility, consequently decreasing the risk of eschars or deep vein thrombosis associated with prolonged bed stay [26].

Moreover, multiple publications have investigated the variation of the vascular endothelial growth factor (VEGF) in connection to NPWT [27,28,29]. When NPWT was applied, local VEGF expression significantly increased in both chronic and acute (traumatic) wounds. A concentration gradient was described by Erba and colleagues, with the tissue near the wound surface, alongside the graft, expressing the highest level [30]. Better vascularization and improved wound healing were the outcomes of the NPWT group’s gradient of expression.

Fibroblast growth factor (FGF) 2 (b-FGF), a powerful mitogen that induces an angiogenic phenotype, was also studied in the setting of NPWT application [31]. A total of 48 h after NPWT treatment, a higher FGF-2 expression was observed in murine fibroblast models, assisting in processes such as neovascularization and fibrosis [32].

Furthermore, dressings impregnated with silver nanoparticles are efficient instruments to prevent wound infection. The mechanisms of silver’s antibacterial effect on sensitive species are diverse. This process starts with silver binding to cell membranes, leading to intracellular absorption by endocytic vacuoles and phagocytosis [33]. The inactivation of membrane-related enzymes causes the denaturation of the bacterial cell envelope and its functional capacity to regulate the inward diffusion of nutrients [34]. Membrane damage, shown as pitting and increased permeability, has been recognized as a precursor to lethality. The potential of silver to disrupt essential intracellular enzyme systems by affecting trace metals and electrolytes likely accounts for most of its intracellular effects. The accumulated damage leads to deficient respiratory pathways and RNA and DNA replication, resulting in bacterial death. Moreover, silver nanoparticles seem to also have a role in countering antibiotic resistance. These effects can be visible even from small concentrations of silver ions, as low as 1 ppm [35].

In addition, the research indicates that dressings infused with silver nanoparticles promote wound healing [36]. They are known to hasten wound closure by interacting with keratinocytes and fibroblasts, causing wound contraction and healing. Silver particles have also been shown to modulate cytokine levels by inhibiting neutrophils and decreasing the quantity of reactive oxygen species [37].

Regarding the cellular influence and macrophage immunomodulation produced by ionic silver (Ag+), Varela et al. reported that there was an impressive increase in TGF-cytokine secretion after interaction with silver-containing dressings [38]. Hesketh et al. reported that ionic silver molecules trigger macrophage immunomodulation toward the M2-state, potentially M2a or M2c, two pro-angiogenic subtypes with important roles in wound healing. TGF secretion, which has profibrotic properties and is linked with the M2a and M2c subsets of M2 macrophages, is also significantly increased by silver-containing dressings [39]. Additionally, in the presence of silver-containing materials, macrophages consume slightly less glucose, demonstrating that these macrophages are polarizing on a continuous spectrum toward M2-macrophages, as an increased expression of glucose transporters (primarily GLUT1) on the membrane and higher rates of glucose consumption have been associated with pro-inflammatory M1 macrophages [40].

Given the fact that AgNPs can help eliminate bacteria and convert macrophages into a wound-healing population, they are a viable treatment for chronic wounds, with positive effects on inflammatory response inhibition and proper cicatrization [41].

Overall, the combination of NPWT and silver nanoparticle sheets proves a synergistic and beneficial effect on wound healing and graft integration. As Khansa et al. reported, infected diabetic foot ulcers were proven to heal more quickly with the use of silver-coated sponges instead of plain ones, as they reduce the bacterial growth of biofilm-causing species (Pseudomonas aeruginosa and Staphylococcus aureus, even MRSA) [42,43]. The synergistic effects of silver and NPWT can be achieved by applying a silver layer underneath a standard polyurethane sponge or by utilizing a polyurethane sponge coated with silver [44].

Moreover, in the study conducted by Hanh et al., according to the tissue culture findings, a significantly lower rate of bacterial growth was noted in the group of patients that received NPWT+silver dressings, compared to the control group. The much lower incidence of bacterial colonization further supports the effectiveness of the inclusion of AgNP-coated dressings in NPWT systems [45]. The study showed better outcomes in terms of infection when using silver-impregnated NPWT, compared to the standard one [46].

As highlighted by Sapino et al. in their comparative study, there was a difference in graft integration between VAC patients and non-VAC patients, with rates of 92% compared to 72% [47]. Mo et al. observed a slightly lower difference in graft outcome: 86.7% integration for NPWT-treated patients, compared to 74.1% for those with tie-over bolsters [48]. In our study, we observed a 99.62% graft integration at the 14-day threshold postoperatively for patients benefiting from the NPWT-Ag+ combination, compared to 93.41% for patients treated with silver nanoparticle dressings alone. Therefore, we observed that an improvement in graft take resulted from the use of silver nanoparticle sheet in conjunction with NPWT. Another study conducted by Blume et al. showed a significant difference in graft outcome at the 5-day threshold postoperatively, with values of 97% for NPWT-treated patients, compared to 84% for conventional therapy groups [49]. This further reinforces the hypothesis that NPWT contributes to enhanced early graft integration, as highlighted by our results.

Patients with diabetes mellitus present with multiple alterations of peripheral homeostasis, such as improper blood supply, caused by microangiopathy and vegetative neuropathy, and predisposition to chronic ulcer infection, often with resistant microorganisms [50,51,52]. A study conducted by Wu et al. compared graft integration rates in diabetic patients, showing a skin graft survival rate of 100% for the NPWT-treated group, contrasting with 76% for those healed with conventional moist dressings [53]. Another study by Bordianu et al. showed complete graft integration for 56 out of 63 cases of grafted diabetic foot ulcerations with the use of NPWT [54]. Furthermore, Ramanujam et al. demonstrated an increased healing time for diabetic patients (5.74 weeks compared to 3.75 weeks) [7]. Our data suggest that there is a benefit in using NPWT+AgNPs for diabetic patients, with an integration rate of 99.67% at 2 weeks compared to 91.29% for the other lot, and a stronger effect size of −1.23 for diabetic patients compared to 0.48 for nondiabetics.

Congestive heart failure and other cardiovascular comorbidities may also influence the process of graft take. Turissini et al. demonstrated a higher probability of skin graft failure in patients suffering from congestive heart failure [55]. On the other hand, Lim et al. established that 19 out of 41 patients suffering from ischemic heart disease achieved complete wound healing under NPWT [56]. Our data also suggest that the combination of NPWT and silver nanoparticle dressings improved skin graft integration in all patients, with an integration rate of 99.29% for patients without heart disease and 99.8% for patients with heart disease (compared to 93.75%, respectively 93.33% for patients treated with silver dressings).

Smaller grafts generally adhere better to the wound bed, while larger grafts are more prone to decreased graft integration and local complications, as stated by Zhao et al. [57]. Another study by Liao et al. showed higher integration rates for smaller grafts (92% vs. 82%) [58]. Furthermore, Nakamura et al. compared grafts of similar medium sizes (mean of 391.2 cm^2^ for tie-over and 254.6 cm^2^ for NPWT), and they found an improvement in graft take for those treated with NPWT [59]. Our data align with this hypothesis, showing better results for smaller grafts, ranging from 100% and 98.57% for those treated with NPWT+AgNPs and silver dressings, respectively, to 99.38 and 80% for large grafts managed likewise. It is also significant to underline the higher effect size of NPWT when used on larger grafts.

From a pathophysiological standpoint, leg ulcerations are characterized by increased leukocyte infiltration and endothelial cell permeability, as well as elevations in inflammatory cytokines, matrix metalloproteinases (MMPs), reactive oxygen and nitrogen species, iron deposition, and tissue metabolites [60]. A study investigating the use of NPWT after lower limb ulceration debridement reported improvement in graft adherence compared to conventional dressing (99.2% for NPWT and 89.7% for conventional dressing) [61]. Moreover, Kantak et al. stated that NPWT also showed its benefits in burn patients, with a median graft integration of 97% [62]. Our analyzed data suggest a similar phenomenon, with higher graft takes for acute surgical wounds compared to chronic ulcerations. Nevertheless, the combination of NPWT+AgNPs seems to offer a greater benefit in the case of debrided ulcerations.

CRP is an acute response protein, mainly produced in the liver, although it may originate from other tissues in certain conditions. The CRP content in the blood rises sharply from 0.8 mg/L to 600–1000 mg/L during the acute-phase response to inflammation, peaking after around 48 h. CRP has a half-life of around 19 h, and once the trigger for the enhanced production stops, the concentration in the blood quickly recovers to baseline levels [63]. High CRP readings more accurately represent inflammation and/or tissue damage than other markers such as plasma viscosity and erythrocyte sedimentation rate, even if there is no apparent association between the two and the severity of the disease [64]. A study by Legendre et al. compared the CRP blood levels of 41 individuals with chronic venous leg ulcers with those of a control group without ulcers and demonstrated a positive correlation between CRP and wound healing. Individuals who experienced various wound problems (for example, an infection) had CRP levels that were, on average, greater (~35 mg/L) than those participants who did not experience any issues (~9 mg/L). It was believed that a concentration greater than 15 mg/mL indicates wound inflammation [65].

A study by Rashid et al. demonstrated a decrease in C reactive protein levels after applying NPWT (78% of patients) [66]. Another study by Liu et al. showed declining CRP levels from 66.4 mg/l to 10.4 mg/l after a mean NPWT application time of 13 days [67]. Accordingly, our data suggest a decrease in CRP levels after NPWT treatment, with an accentuated drop at the 10-day threshold, in line with an accelerated graft integration observed for patients that benefitted from the combination of NPWT and silver nanoparticle dressings.

The strengths of this study are represented by the innovative combination of NPWT dressings with silver nanoparticle dressings, mutually enhancing their antimicrobial and anti-inflammatory effects, and showing great results in promoting graft integration for both acute and chronic wounds. Furthermore, we present an improved management approach to chronic ulcers larger than 150 cm^2^. Taking into consideration that there is no other viable reconstruction method for this pathology, our proposal is cost-efficient and helps decrease the burden on the medical care system. Consequently, we are currently developing a prospective study focusing on the quality of life of patients with this condition. Subsequently, our goal is to update the protocols currently in use for the treatment of skin ulcers. However, it is important to note that this study faced limitations due to pandemic restrictions, resulting in fewer cases during the selected period and a decrease in the accessibility of patients to public health institutions. Another limitation of this study is the absence of blinding.

**Figure 3:**
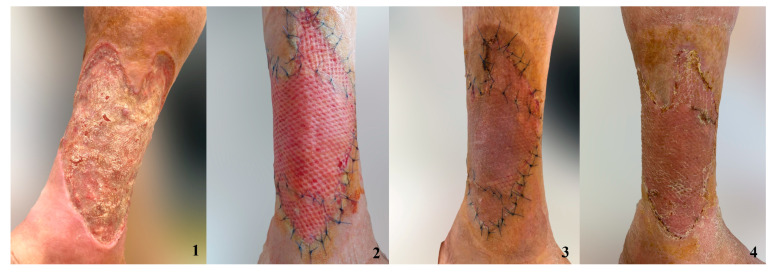
Grafted chronic ulceration treated with the combination of NPWT and AgNPs. 1. Ulcer 2. Skin graft after 7 days 3. Skin graft after 10 days 4. Skin graft after 14 days.

**Figure 4:**
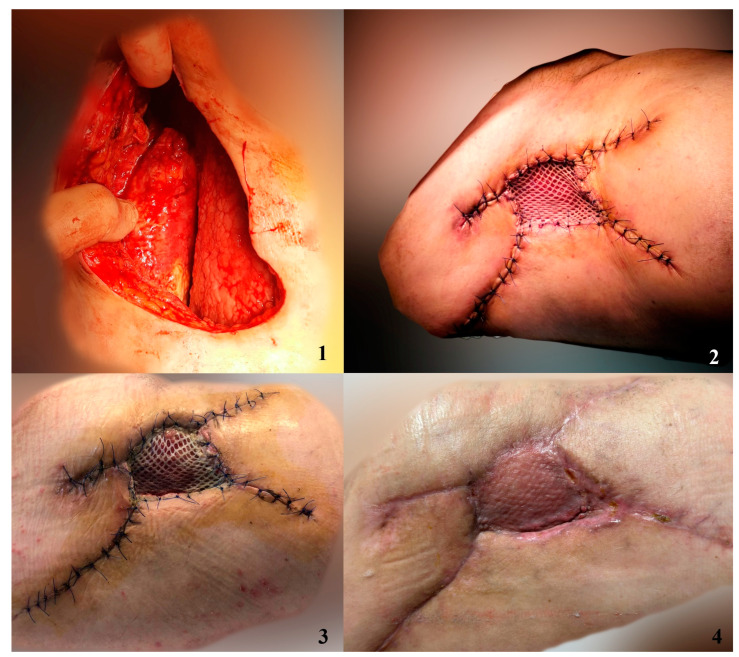
Patient presented with Morel-Lavallée lesion. After debridement and progressive closure of subcutaneous degloving, we covered the remaining defect with a skin graft dressed with NPWT+AgNPs. 1. Debrided lesion 2. Skin graft imediately after surgery 3. Skin graft after 7 days 4. Skin graft after 14 days.

**Figure 5:**
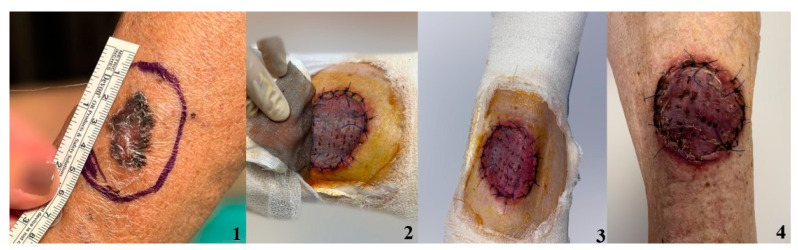
Defect after melanoma excision covered with STSG and dressed with silver nanoparticle sheet. 1. Cutaneous tumor prior to excision; 2. Skin graft after 7 days; 3. Skin graft after 10 days 4. Skin graft after 14 days.

**Figure 6:**
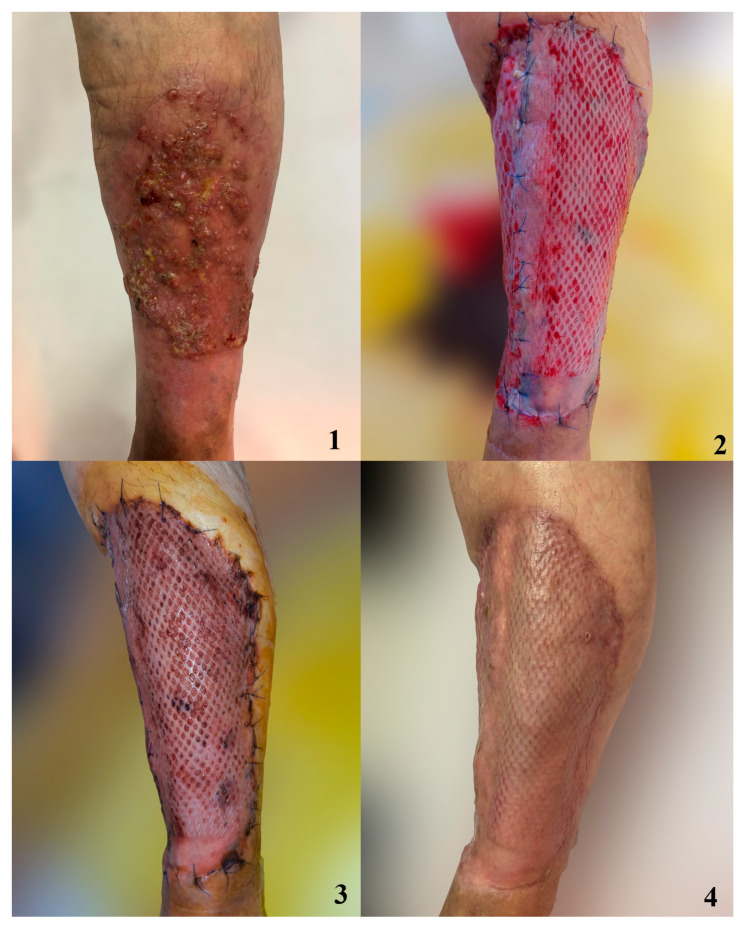
Patient with extensive basal cell carcinoma of the thigh following a chronic ulceration. 1. Preoperative aspect of tumor 2. Graft managed with compressive dressing at 7 days postoperatively. 3. Graft at 10 days postoperatively. 4. Graft at 14 days postoperatively.

In order to better visualize the graft integration using the studied dressing methods, we have included photos depicting the process of graft healing (Figure 3, Figure 4, Figure 5 and Figure 6).

## 5. Conclusions

Many factors influence the postoperative evolution of a split-skin graft, from general factors such as patient status and comorbidities to local factors such as mechanical shearing forces, lifting of the graft from the wound bed, or infection. Postoperative dressings address local factors to further increase graft integration.

The results of our study pointed out improvements in the treatment and care of grafted patients using NPWT combined with a silver nanoparticle sheet compared to silver nanoparticle dressings secured with tie-over bolsters. The most notable benefits of this novel dressing method are better graft integration as well as decreased levels of systemic inflammatory markers.

## Figures and Tables

**Table 1:** Distribution of patients.

		Silver Nanoparticle Sheet	NPWT+AgNPs	Total
Defect origin	Postexcisional defect	17	11	28
	Ulceration debridement	24	28	52
Graft area (cm^2^)	<150 cm^2^	14	11	25
	150–500 cm^2^	22	20	42
	>500 cm^2^	5	8	13
Cardiovascular comorbidities	No	8	14	22
	Yes	33	25	58
Diabetes mellitus	No	10	9	19
	Yes	31	30	61

**Table 2:** Graft integration depending on type of dressing used.

		Mean	Std. Deviation	*p*-Value
Graft take after 7 days (%)	Silver nanoparticle sheet	74.88	11.374	<0.001
	NPWT+AgNPs	85.00	8.959	
Graft take after 10 days (%)	Silver nanoparticle sheet	87.20	9.686	<0.001
	NPWT+AgNPs	97.56	4.115	
Graft take after 14 days (%)	Silver nanoparticle sheet	93.41	8.905	<0.001
	NPWT+AgNPs	99.62	1.771	

**Table 3:** Graft evolution depending on cardiovascular comorbidities.

		No CV Disease				CV Disease			
		Mean	Std. Deviation	*p* Value	Effect Size	Mean	Std. Deviation	*p* Value	Effect Size
Graft take after 7 days (%)	Silver nanoparticle sheet	79.38	10.501	0.718	−0.16246	73.79	11.459	<0.001	−1.35267
	NPWT+AgNPs	81.07	10.411			87.20	7.371		
Graft take after 10 days (%)	Silver nanoparticle sheet	90.63	9.039	0.064	−0.86838	86.36	9.785	<0.001	−1.52861
	NPWT+AgNPs	96.43	4.972			98.20	3.500		
Graft take after 14 days (%)	Silver nanoparticle sheet	93.75	7.906	0.092	−1.07499	93.33	9.242	<0.001	−0.92157
	NPWT+AgNPs	99.29	2.673			99.80	1.000		

**Table 4:** Graft evolution depending on diabetes mellitus type 2.

		No Diabetes				Diabetes Mellitus			
		Mean	Std. Deviation	*p* Value	Effect Size	Mean	Std. Deviation	*p* Value	Effect Size
Graft take after 7 days (%)	Silver nanoparticle sheet	84.00	8.756	0.098	−0.8043	71.94	10.621	<0.001	−1.17855
	NPWT+AgNPs	91.11	8.937			83.17	8.251		
Graft take after 10 days (%)	Silver nanoparticle sheet	96.00	4.595	0.304	−0.4871	84.35	9.196	<0.001	−1.82809
	NPWT+AgNPs	98.33	5.000			97.33	3.880		
Graft take after 14 days (%)	Silver nanoparticle sheet	100.00	0.000	0.347	0.4859	91.29	9.307	<0.001	−1.23936
	NPWT+AgNPs	99.44	1.667			99.67	1.826		

**Table 5:** Graft evolution depending on graft area.

		<150 cm^2^			150–500 cm^2^			>500 cm^2^		
		Mean	*p* Value	Effect Size	Mean	*p* Value	Effect Size	Mean	*p* Value	Effect Size
Graft take after 7 days (%)	Silver nanoparticle sheet	84.29	0.068	−0.77274	72.05	0.000	−1.49177	61.00	0.000	−3.50173
	NPWT+AgNPs	89.55			86.25			75.63		
Graft take after 10 days (%)	Silver nanoparticle sheet	95.00	0.004	−1.21345	85.23	0.000	−1.81813	74.00	0.000	−4.34509
	NPWT+AgNPs	99.55			97.75			94.38		
Graft take after 14 days (%)	Silver nanoparticle sheet	98.57	0.218	−0.46041	93.18	0.003	−0.99211	80.00	0.002	−4.90153
	NPWT+AgNPs	100.00			99.50			99.38		

**Table 6:** Graft evolution depending on defect origin.

		Postexcisional				Ulceration Debridement			
		Mean	Std. Deviation	*p* Value	Effect Size	Mean	Std. Deviation	*p* Value	Effect Size
Graft take after 7 days (%)	Silver nanoparticle sheet	82.65	7.729	0.000	−1.60545	69.38	10.354	0.000	−1.3342
	NPWT+AgNPs	93.18	4.045			81.79	8.302		
Graft take after 10 days (%)	Silver nanoparticle sheet	93.53	5.524	0.000	−1.49319	82.71	9.553	0.000	−1.90856
	NPWT+AgNPs	100.00	0.000			96.61	4.524		
Graft take after 14 days (%)	Silver nanoparticle sheet	98.82	2.811	0.104	−0.53342	89.58	9.771	0.000	−1.45283
	NPWT+AgNPs	100.00	0.000			99.46	2.081		

**Table 7:** Variation of CRP depending on the type of dressing used.

		Mean	Std. Deviation	*p*-Value
CRP before graft (mg/dL)	Silver nanoparticle sheet	4.020	2.8149	0.868
	NPWT+AgNPs	3.915	2.7547	
CRP after 7 days (mg/dL)	Silver nanoparticle sheet	38.02	5.985	0.481
	NPWT+AgNPs	36.97	7.235	
CRP after 10 days (mg/dL)	Silver nanoparticle sheet	11.473	6.5328	0.001
	NPWT+AgNPs	7.267	3.9482	
CRP after 14 days (mg/dL)	Silver nanoparticle sheet	4.129	2.4921	0.145
	NPWT+AgNPs	3.338	2.3040	

## Data Availability

The raw data supporting the conclusions of this article will be made available by the authors on request.
